# Identification of a robust multitarget protein panel for Parkinson’s disease via absolute quantification and large-scale external replication

**DOI:** 10.1093/braincomms/fcag282

**Published:** 2026-07-16

**Authors:** Ho-Won Lee, Youngtae Choi, Shinrye Lee, Jung-Eun Kim, Min-Tae Jeon, Myungjin Jo, Gyuri Park, Jin-Sung Park, Do-Geun Kim, Mookyung Cheon, Hyung-Jun Kim

**Affiliations:** Department of Neurology, Kyungpook National University Chilgok Hospital, Daegu 41404, Republic of Korea; Department of Neurology, School of Medicine, Kyungpook National University, Daegu 41944, Republic of Korea; Brain Science & Engineering Institute, Kyungpook National University, Daegu 41944, Republic of Korea; Dementia Research Group, Korea Brain Research Institute (KBRI), Daegu 41062, Republic of Korea; Dementia Research Group, Korea Brain Research Institute (KBRI), Daegu 41062, Republic of Korea; Department of Neurology, Kyungpook National University Chilgok Hospital, Daegu 41404, Republic of Korea; Dementia Research Group, Korea Brain Research Institute (KBRI), Daegu 41062, Republic of Korea; Dementia Research Group, Korea Brain Research Institute (KBRI), Daegu 41062, Republic of Korea; Dementia Research Group, Korea Brain Research Institute (KBRI), Daegu 41062, Republic of Korea; Department of Neurology, Kyungpook National University Chilgok Hospital, Daegu 41404, Republic of Korea; Department of Neurology, School of Medicine, Kyungpook National University, Daegu 41944, Republic of Korea; Brain Science & Engineering Institute, Kyungpook National University, Daegu 41944, Republic of Korea; Dementia Research Group, Korea Brain Research Institute (KBRI), Daegu 41062, Republic of Korea; Dementia Research Group, Korea Brain Research Institute (KBRI), Daegu 41062, Republic of Korea; Dementia Research Group, Korea Brain Research Institute (KBRI), Daegu 41062, Republic of Korea; Graduate School, DGIST, Daegu 42988, Republic of Korea

**Keywords:** Parkinson’s disease, neuroinflammation, plasma biomarkers, interleukin-17C, urokinase plasminogen activator

## Abstract

This study aimed to identify and validate a robust, generalizable panel of plasma protein biomarkers to improve diagnostic precision in Parkinson’s disease. We analysed plasma samples from 12 patients with [^18^F]-FP-CIT PET-confirmed Parkinson’s disease and 15 healthy controls using the Olink Target 96 Inflammation Panel to identify differentially expressed proteins. Candidate biomarkers were subsequently validated through absolute quantification using Luminex and Olink Flex platforms in an independent cohort of 46 patients with Parkinson’s disease and 33 amyloid-negative and cognitively normal control participants. To assess generalizability, the findings were replicated across multiple heterogeneous populations using large-scale datasets from the UK Biobank and Global Neurodegeneration Proteomics Consortium (GNPC) cohorts. Markers of neurodegeneration [neurofilament light chain (NfL)] and Alzheimer’s disease [phosphorylated tau (pTau181), amyloid β [Aβ]42, Aβ40] co-pathology were measured using the single molecule array platform. Our analyses revealed elevated levels of interleukin (IL)-10 and IL-17C and reduced levels of urokinase plasminogen activator (uPA) and neurotrophin-3 (NTF3) in patients with Parkinson’s disease. These findings were confirmed in the validation cohort. Multitarget models demonstrated superior diagnostic performance over individual markers, with the combination of IL-17C and uPA achieving the highest discrimination (area under the curve = 0.780). External validation in the UK Biobank and GNPC datasets confirmed consistent directional changes of three candidates (IL-17C, NTF3 and uPA), reinforcing the biological relevance of these markers. Notably, while NfL levels were significantly elevated in Parkinson’s disease, no significant differences were observed for pTau181 levels or Aβ42/Aβ40 ratios. These findings identify a specific plasma protein panel, particularly the combination of IL-17C and uPA, as a robust and generalizable diagnostic signature that captures fundamental pathophysiological aspects of Parkinson’s disease and enhances diagnostic precision alongside established biomarkers.

## Teaser

Lee *et al*. report that combining plasma interleukin-17C and urokinase plasminogen activator in a multitarget panel improves diagnostic discrimination for Parkinson’s disease compared with individual markers alone. These biomarkers, identified using proximity extension assay technology and validated through absolute quantification, showed consistent directional changes across independent large-scale cohorts, supporting their potential biological relevance.

## Graphical Abstract

**Graphical Abstract:**
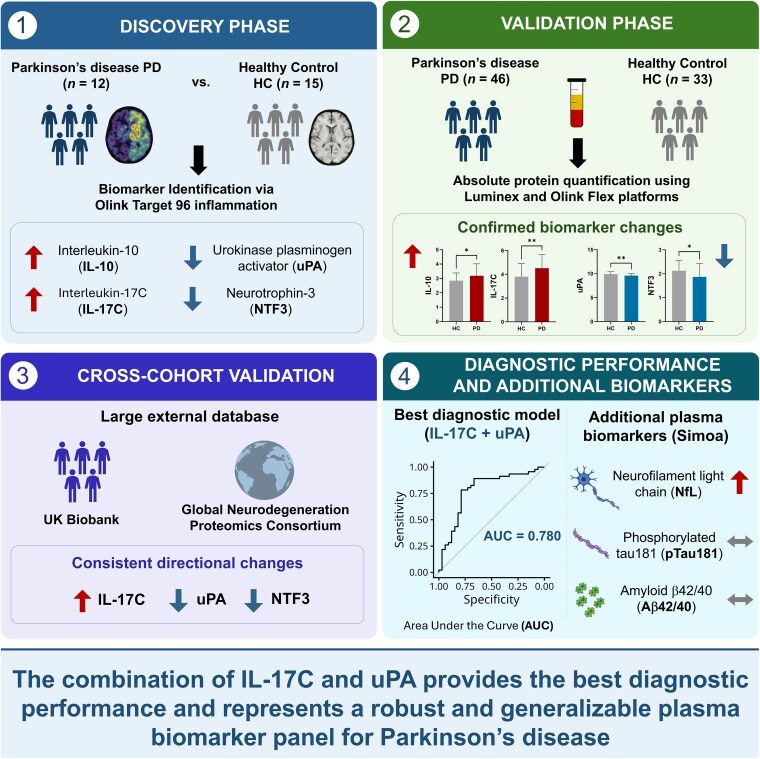

## Introduction

Parkinson’s disease represents a major global health challenge. This neurodegenerative disorder manifests with both motor and non-motor symptoms due to the loss of dopaminergic neurons in the substantia nigra pars compacta.^1^ Although the mechanisms underlying Parkinson’s disease remain incompletely understood, substantial evidence underscores the central role of neuroinflammation in the pathogenesis of Parkinson’s disease, involving both central and peripheral inflammatory responses in the development and progression of the disease.^2,3^

Systematic meta-analyses of over 150 studies have demonstrated that compared with healthy controls (HCs), patients with Parkinson’s disease exhibit significantly elevated levels of inflammatory biomarkers, including cytokines, such as interleukin (IL)-6, tumor necrosis factor-α and IL-1β, as well as C-reactive protein and various chemokines, in both peripheral blood and cerebrospinal fluid.^3^ These alterations indicate a distinctive immune profile and ongoing inflammatory processes in Parkinson’s disease but also correlate with clinical features.^3^ The results of several studies indicate that using a combination of inflammatory markers improves the ability to differentiate patients with Parkinson’s disease from HC, highlighting the potential role of these marker combinations in the diagnosis and monitoring of this disease.^4-6^ Nonetheless, due to the variability in assay techniques and patient demographics, further research is essential to standardize biomarker evaluations and determine their clinical value in Parkinson’s disease.

The development of Olink Proximity Extension Assay (PEA) technology has advanced protein biomarker discovery, particularly for inflammatory factors.^7-9^ This method combines antibody-based immunoassays with PCR amplification, enabling highly specific and sensitive multiplex protein analysis with only small amounts of samples.^10^ Recent studies utilizing PEA technology have identified promising inflammatory biomarkers in CSF and blood samples from patients with Parkinson’s disease, distinguishing them from HC and individuals with other neurodegenerative conditions, including atypical parkinsonism and Alzheimer’s disease.^11-17^ While some of these studies showed superior performance compared to existing known biomarkers, multivariate analysis utilizing multiple proteins was not conducted due to the limitations of relative quantification inherent in this technology.

Large-scale proteomics resources, including the Global Neurodegeneration Proteomics Consortium (GNPC) and the UK Biobank, now provide extensive plasma proteomic datasets from patients with Parkinson’s disease and control participants.^18,19^ These resources enable the validation of candidate biomarkers identified in smaller discovery cohorts across diverse populations and clinical settings, supporting the robustness and generalizability of findings.

To identify novel biomarkers for Parkinson’s disease, we employed the Olink Target 96 Inflammation Panel to comprehensively profile clinically relevant proteins in a discovery cohort. Subsequently, we validated the absolute concentrations of candidate biomarkers using complementary immunoassay platforms (Luminex and Olink Flex) in an independent cohort of patients with Parkinson’s disease. We also assessed clinical utility and performed cross-cohort validation using external datasets from the GNPC and UK Biobank. This integrated approach aimed at the robust identification, validation and clinical characterization of potential plasma biomarkers for Parkinson’s disease.

## Materials and methods

### Study population and ethics approval

All participants visited the Neurodegenerative Disease Center of Kyungpook National University Chilgok Hospital (KNUCH) between 2020 and 2023 for their evaluation. Informed consent was obtained from patients and their caregivers before study participation. This study was approved by the institutional review board of the KNUCH (KNUMC-2023-01-036-001).

We enrolled patients with Parkinson’s disease who met the UK Parkinson’s Disease Society Brain Bank clinical diagnostic criteria.^20^ These diagnoses were confirmed using [^18^F]N-(3-fluoropropyl)-2β-carboxymethoxy-3β-(4-iodophenyl)nortropane ([^18^F]-FP-CIT) PET images showing reduced dopaminergic uptake in the posterior putamen. The HC group included participants between 55 and 85 years of age with no known health factors negatively influencing cognitive performance and with age- and education-adjusted Mini-Mental State Examination (MMSE) scores of at least the average minus one standard deviation.^21^ The HC group underwent [^18^F]-florbetaben (amyloid) PET of the brain to exclude participants with potential preclinical stage amyloid pathology.

### Clinical assessment

Specialized neurologists evaluated and diagnosed all study participants. Neuropsychological data, including the Korean MMSE (K-MMSE) and clinical dementia rating (CDR) scores, were obtained for all participants. Severity of Parkinson’s disease symptoms was assessed using the Unified Parkinson’s disease rating scale (UPDRS), Hoehn & Yahr scale and Schwab and England Activities of Daily Living (SEADL).

### Sample collection and preparation

Peripheral blood (10 ml) was collected from each patient in EDTA-coated tubes (BD Vacutainer®, Becton Dickinson, USA). The tubes were kept in an upright position at room temperature for 30 min to allow complete mixing with the anticoagulant. Subsequently, the samples were centrifuged at 3000 rpm for 10 min at room temperature. The resulting plasma was carefully aliquoted into sterile 1.5 ml microcentrifuge tubes and immediately stored at −80°C until further use.

### Proteomic analysis

The Olink Inflammation 96 Panel (Olink Analysis Service, Uppsala, Sweden) was used to analyse plasma samples from HC (*n* = 15) and patients with Parkinson’s disease (*n* = 12). This panel measures the normalized protein expression (NPX) levels of 92 proteins involved in inflammatory and immune responses. Samples and protein assays were filtered based on the limit of detection and the deviation from the control median NPX value. After filtering, 75 protein assays with valid data in >75% of the samples were retained.

### Measurement of plasma IL-17C, IL-10, NTF3 and uPA concentrations

To validate and further investigate the identified targets [IL-17C, IL-10 and neurotrophin-3 (NTF3)], the Olink Flex Panel was applied to an independent cohort of HC (*n* = 46) and patients with Parkinson’s disease (*n* = 33). The concentration of urokinase plasminogen activator (uPA), one of the initially identified targets, was independently quantified using the Human Premixed Multi-Analyte Luminex Assay Kit (Catalog No. LXSAHM, R&D Systems, USA) according to the manufacturer’s protocol.

### Measurement of plasma pTau181, Aβ40, Aβ42 and NfL concentrations

Plasma phosphorylated tau (pTau181), amyloid β (Aβ)40, Aβ42 and neurofilament light chain (NfL) concentrations were measured using the single molecule array (Simoa) platform. We measured pTau181 levels using the pTau181 V2 Simoa Advantage Assay (QTX-103714), with both calibrators and samples processed in duplicate. The concentrations of Aβ40 and Aβ42 were determined using the Neurology 3-Plex A advantage kit (QTX-101995), whereas NfL levels were assessed with the NF-light™ V2 Advantage Kit (QTX-101995; Quanterix, Billerica, MA, USA).

### Statistical analysis, including logistic regression analysis

Differentially expressed proteins (DEPs) between the Parkinson’s disease and HC groups were identified using the Mann–Whitney U test (*P* < 0.05) on the NPX values and further confirmed by ANCOVA adjusting for age and sex. We applied logistic regression to predict Parkinson’s disease diagnosis using NPX protein levels. Univariate models were first tested for individual biomarkers, followed by models adjusted for age and sex. Since NPX values are on a log_2_ scale, differences between two proteins, which are equivalent to log_2_ ratios of their concentrations, were also used to assess the combined diagnostic performance of biomarker pairs. Model performance was evaluated using accuracy, sensitivity, specificity and the area under the receiver operating characteristic (ROC) curve (AUC). Differences between AUCs were tested using DeLong’s method, and the model fit was compared using *P*-values from DeLong’s test and Akaike Information Criterion (AIC) values relative to the baseline model. Leave-one-out cross-validation and 5-fold cross-validation were used for validation. A summary of the statistical tests and corresponding assumptions applied across analyses is provided in Supplementary Table 1. All analyses were performed using R version 4.4.3.

### External validation using GNPC and the UK Biobank Proteomic Database

GNPC cohort analysis: External validation was performed using plasma proteomics data from the GNPC,^18,22^ measured using the SOMAscan platform. To maximize phenotypic clarity regarding Parkinson’s disease-related inflammation, we implemented more stringent exclusion criteria than those used in previous GNPC studies. To rule out potential diagnostic shifts over time, only the last available visit for each participant was included. Both Parkinson’s disease and HC groups were rigorously screened to remove individuals with any history of other neurodegenerative diseases, stroke, transient ischaemic attack, traumatic brain injury or cancer—conditions associated with considerable systemic inflammation or brain injury. Furthermore, HC were restricted to those without metabolic or psychiatric disorders (identified via demographic data and ICD-10 codes) and were required to have a CDR score of 0 and cognitively normal MMSE/MoCA performance. Statistical differences in IL-10, IL-17C, NTF3 and uPA levels were evaluated using ANCOVA, adjusted for age, sex and cohort, consistent with prior GNPC analytical frameworks.^22,23^

UK Biobank analysis: Validation was further extended to the UK Biobank using the Olink Explore 3072 platform.^19,24^ Given the UK Biobank’s relatively young distribution compared to typical neurodegenerative cohorts, both Parkinson’s disease and HC groups were restricted to participants aged 60 years or older. Parkinson’s disease cases were identified using broadened criteria, which included diagnoses made after the initial blood collection, to encompass both clinical and prodromal stages. In contrast, HC were strictly curated by excluding individuals with neurological, metabolic, inflammatory, or mental disorders based on ICD-10 codes and clinical metadata. Consistent with the GNPC analysis, group differences in the four target proteins were evaluated using ANCOVA, adjusted for age and sex.

## Results

### Participant demographics and clinical characteristics

The discovery cohort comprised 12 individuals diagnosed with Parkinson’s disease and 15 HCs. Both groups had similar mean ages (70.8 ± 8.6 versus 70.9 ± 5.6 years, *P* = 0.961) and sex distributions (Parkinson’s disease: 58.3% male, HC: 46.7% male, *P* = 0.830). Notably, patients with Parkinson’s disease had lower K-MMSE scores than HC (25.1 ± 4.4 versus 28.9 ± 1.2, *P* = 0.016; Table 1). The validation cohort comprised 46 patients with Parkinson’s disease and 33 HCs, with no significant age difference observed (72.3 ± 7.9 versus 72.5 ± 4.9 years, *P* = 0.929). However, the Parkinson’s disease group had a significantly higher proportion of male participants than the HC group (60.9% versus 18.2%, *P* < 0.001). Cognitive impairment was more severe in the Parkinson’s disease group (K-MMSE 23.7 ± 5.3 versus 28.5 ± 1.4, *P* < 0.001), and they also exhibited greater functional decline (CDR 0.48 ± 0.32 versus 0.27 ± 0.25, *P* = 0.002). The mean disease duration was 8.0 ± 4.1 years, with the onset of symptoms occurring at an average age of 64.6 ± 9.0 years (Table 1). The diagnoses of all patients with Parkinson’s disease who participated in this study were confirmed through [^18^F]-FP-CIT PET imaging, and it was verified that the participants in the HC group were amyloid PET-negative.

**Table 1:** Demographic and clinical characteristics of study participants

Discovery cohort	Parkinson’s disease (n=12)	HC (n=15)	*P*-value Parkinson’s disease versus HC
Age (year)	70.83 ± 8.64	70.93 ± 5.59	0.961^b^
Gender (F:M)	5:7	8:7	0.830^c^
K-MMSE total score	25.09 ± 4.39	28.86 ± 1.23	0.016^b^
UPDRS Part I	5.00 ± 3.19		
UPDRS Part II	11.45 ± 6.28		
UPDRS Part III	21.82 ± 6.11		
UPDRS Part IV	1.09 ± 2.02		
Modified H&Y	2.32 ± 0.40		
SEADL score	81.82 ± 12.50		

Validation cohort	Parkinson’s disease (n=46)	HC (n=33)	*P*-value Parkinson’s disease versus HC
Age (year)	72.28 ± 7.92	72.45 ± 4.85	0.929^a^
Gender (F:M)	18:28	27:6	<0.001^c^***
Age at symptom on set (years)	64.61 ± 9.02		
Disease duration (years)	7.98 ± 4.13		
K-MMSE total score	23.70 ± 5.30	28.48 ± 1.35	<0.001^a^***
CDR	0.48 ± 0.32	0.27 ± 0.25	0.002^a^**
UPDRS Part I	4.52 ± 2.87		
UPDRS Part II	12.24 ± 6.65		
UPDRS Part III	24.80 ± 9.29		
UPDRS Part IV	1.07 ± 1.87		
Modified H&Y	2.48 ± 0.57		
SEADL score	79.356 ± 14.82		
NfL	36.69 ± 38.02	18.76 ± 10.45	0.004^a^**
pTau181	7.34 ± 12.24	4.70 ± 6.65	0.226^a^
Aβ40	205.89 ± 101.15	180.35 ± 88.05	0.236^a^
Aβ42	9.45 ± 4.52	9.75 ± 5.26	0.794^a^
Aβ42/Aβ40	0.05 ± 0.02	0.06 ± 0.02	0.381^a^

Data are presented as mean ± SD or *n*(%). Biomarker concentrations (NTF3, IL-10, IL-17C and uPA) are expressed in pg/ml. HC, healthy control; K-MMSE, Korean mini-mental state examination; UPDRS, unified Parkinson’s disease rating scale; UPDRS part I, behavior and emotion; UPDRS part II, activities of daily living; UPDRS part III, motor scale; UPDRS part IV, drug complications; modified H&Y, modified Hoehn and Yahr stage. ^a^Welch’s *t*-test. ^b^Mann–Whitney U test. ^c^Chi-squared test.

Final cohort sizes reflect additional *post hoc* exclusions applied to ensure diagnostic and analytical rigor. In the discovery cohort, plasma samples from 20 individuals with an initial clinical diagnosis of Parkinson’s disease were measured using the Olink panel; however, the subsequent application of stringent diagnostic criteria based on [^18^F]-FP-CIT PET imaging resulted in the reclassification of eight individuals to alternative diagnoses; therefore, these participants were excluded from the Parkinson’s disease group. In the validation cohort, one HC sample was excluded because the Olink Flex measurements failed to meet the quality control thresholds. Consequently, only participants who met both diagnostic and assay quality criteria were included in the final analyses.

### Differential protein expression across patients with Parkinson’s disease and HC

**Figure 1:**
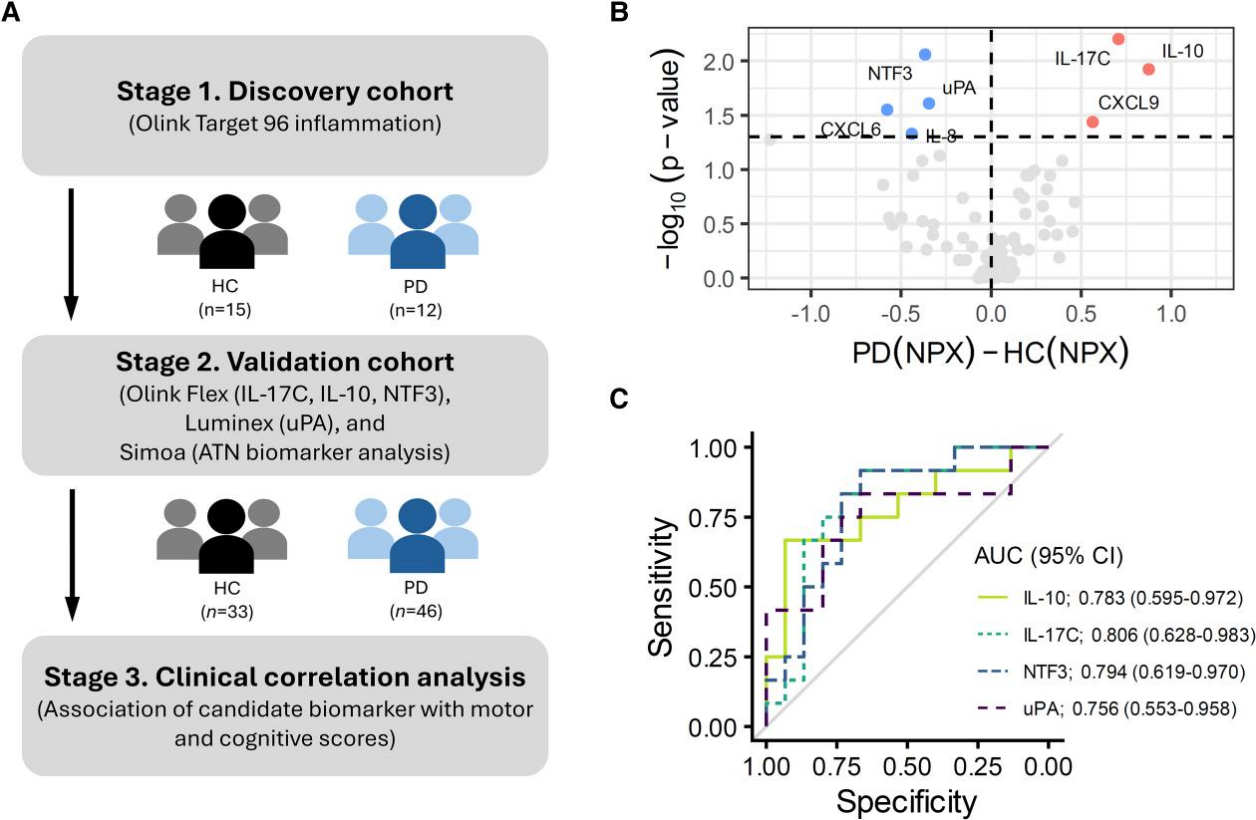
**Identification and diagnostic performance of differentially expressed proteins in Parkinson’s disease compared to HC in discovery cohort.** (**A**) Overview of study design. (**B**) Volcano plot showing DEPs between the Parkinson’s disease (n=12) and HC (n=15) groups in the discovery cohort, based on NPX values. The horizontal dashed line denotes the statistical significance threshold (P=0.05). Each data point represents an individual protein, showing the difference in NPX values between the Parkinson’s disease and HC groups (on the horizontal axis) and its corresponding *P*-value (on the vertical axis). Rank sum statistics (*W*) and *P*-values of Mann–Whitney U test: NTF3: W=143(P=0.009); uPA: W=136(P=0.025); CXCL6: W=135(P=0.028); IL-8: W=131(P=0.047); CXCL9: W=47(P=0.037); IL-10: W=39(P=0.012); IL-17C: W=35(P=0.006). The *P*-values were not adjusted for multiple comparisons. (**C**) ROC curves for logistic regression models using individual DEPs to distinguish Parkinson’s disease (n=12) from HC (n=15) based on NPX values. Abbreviations: AUC, area under the curve; ATN, amyloid, tau and neurodegeneration; CI, confidence interval; CXCL6, C-X-C motif chemokine ligand 6; CXCL9, C-X-C motif chemokine ligand 9; DEP, differentially expressed proteins; HC, healthy control; IL-8, interleukin-8; IL-10, interleukin-10; IL-17C, interleukin-17C; NPX, normalized protein expression; NTF3, neurotrophin-3; PD, Parkinson’s disease; ROC, receiver operating characteristic. Simoa, single molecule array; uPA, urokinase plasminogen activator.

The discovery cohort consisted of 15 HC and 12 patients with Parkinson’s disease, whose plasma proteomes were analysed using the Olink Target 96 Inflammation panel (Fig. 1A). Demographic and clinical characteristics of the discovery cohort are summarized in Table 1. Figure 1B presents a volcano plot illustrating differential protein expression between patients with Parkinson’s disease and HC, based on unadjusted raw NPX values. Using the Mann–Whitney U test, we selected four proteins (two upregulated and two downregulated) ranked by significance (lowest *P*-values) to enable the application of a multitarget-based approach for improved biomarker performance in later analyses. IL-10 and IL-17C were significantly upregulated, whereas NTF3 and uPA were significantly downregulated in Parkinson’s disease. These differences in protein concentrations remained significant in age- and sex-adjusted ANCOVA analyses. Hence, these proteins were prioritized as Parkinson’s disease-specific biomarker candidates.

Next, we applied logistic regression models to classify Parkinson’s disease and HC based on the NPX values of individual DEPs. As shown in Fig. 1C, IL-17C exhibited the highest diagnostic performance among the four selected DEPs, with an AUC of 0.806 (95% confidence interval: 0.628–0.983, calculated using DeLong’s method). The ROC results from age- and sex-adjusted logistic regression models yielded similar AUCs, supporting the robustness of the findings. Notably, IL-17C demonstrated moderate but consistent diagnostic accuracy, underscoring its potential as a biomarker for distinguishing patients with Parkinson’s disease from HC.

Because Olink NPX values represent relative, protein-specific normalized measurements, direct comparisons across different proteins are inherently limited, making it challenging to construct robust multitarget biomarker models based solely on NPX data. Given this methodological constraint and the limited sample size, further validation using absolute protein concentrations, which allow the direct integration of multiple targets within a unified statistical framework, is warranted. Accordingly, subsequent analyses focused on quantitative measurements obtained from the Luminex and Olink Flex platforms, where multivariable logistic regression models were applied to evaluate the combined diagnostic value of these biomarkers in an independent validation cohort.

### Validation of four biomarkers and multitargets using absolute protein concentrations

**Figure 2:**
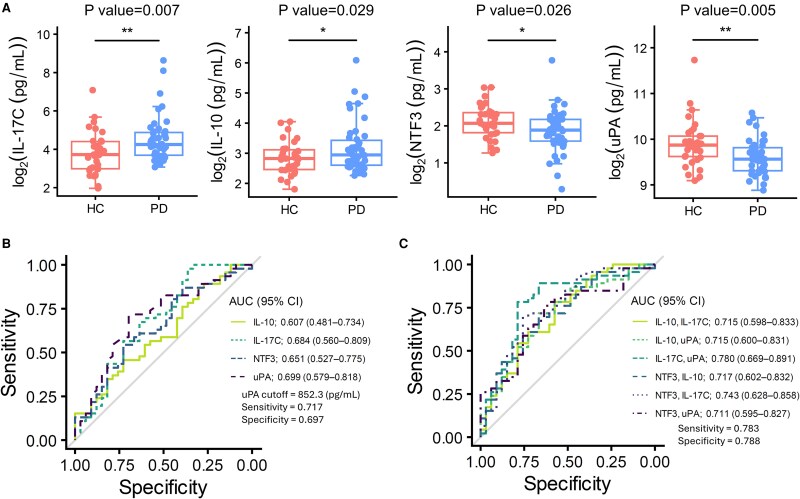
**Absolute plasma concentrations and diagnostic performance of the four protein biomarkers in Parkinson’s disease (*n* = 46) and HC (*n* = 33).** (**A**) Box plots showing the plasma levels of IL-17C, IL-10 and NTF3 measured using the Olink Flex platform and uPA measured using the Luminex platform. Protein concentrations are presented on a log_2_ scale (pg/ml). Each data point represents a sample, showing its log2-transformed absolute plasma concentration. *t* values and *P*-values of Welch’s *t*-test: IL-17C: t=−2.771(P=0.007); IL-10: t=−2.225(P=0.029); NTF3: t=2.265(P=0.026); uPA: t=2.921(P=0.005). (**B**, **C**) ROC curves based on logistic regression using absolute protein concentrations for individual proteins and their multivariable combinations. Abbreviations: AUC, area under the curve; CI, confidence interval; HC, healthy control; PD, Parkinson’s disease; ROC, receiver operating characteristic.

The absolute concentrations of the four candidate proteins IL-17C, IL-10, NTF3 and uPA were measured in a newly assembled validation cohort of ∼79 individuals with Parkinson’s disease and HC (Fig. 1A). The demographic and clinical characteristics of the validation cohort are summarized in Table 1. All four proteins demonstrated significant differences between the two groups, consistent with our previous findings (Fig. 2A and Table 1). However, overall biomarker performance based on absolute concentrations was notably lower than that in the earlier analyses (Fig. 2B and C and Table 2). Among individual proteins, uPA, measured using the Luminex platform, showed the highest diagnostic performance with an AUC of 0.699 (95% confidence interval: 0.579–0.818). When multivariate logistic regression was applied, the linear predictors of the combinations IL-17C with uPA and IL-17C with NTF3 yielded improved AUC values of 0.780 and 0.743, respectively, outperforming all single protein markers. These results confirmed that the use of multitargets enhances the biomarker performance (Table 2). To further assess model robustness and potential overfitting, additional leave-one-out cross-validation and 5-fold cross-validation analyses were performed, showing consistent diagnostic performances across validation strategies (Table 2). Nonetheless, the diagnostic utility of the four selected proteins, originally identified using the Olink Target 96 Inflammation panel, remained limited in this validation cohort. Pairwise correlation analyses of the four proteins showed minimal correlations among the validated biomarkers, except for a significant but modest association between NTF3 and uPA (r = 0.311, *P* = 0.005). By contrast, other pairings, such as IL-10 and IL-17C (*r* = 0.140, *P* = 0.218) or IL-17C and uPA (*r* = −0.084, *P* = 0.463), showed no significant correlation, supporting the appropriateness of multivariable logistic regression modeling (Supplementary Fig. 1).

**Table 2:** Differential expression and diagnostic performance of protein biomarkers in the validation cohort

	Unadjusted	Age-adjusted	Unadjusted		
	log_2_FC (*P*-value^a^)	log_2_FC (*P*-value^b^)	AUC (95% CI^c^)	LOOCV AUC ± SE^d^	5-fold CV AUC ± SD^e^
IL-17C	0.715 (0.007**)	0.713 (0.008**)	0.684 (0.560–0.809)	0.684 ± 0.064	0.683 ± 0.041
IL-10	0.343 (0.029*)	0.339 (0.035*)	0.607 (0.481–0.734)	0.607 ± 0.065	0.606 ± 0.020
NTF3	−0.257 (0.026*)	−0.257 (0.034*)	0.651 (0.527–0.775)	0.651 ± 0.064	0.650 ± 0.039
uPA	−0.307 (0.005**)	−0.309 (0.003**)	0.699 (0.579–0.818)	0.699 ± 0.062	0.701 ± 0.016
NTF3, IL-10			0.717 (0.602–0.832)	0.719 ± 0.064	0.717 ± 0.029
NTF3, IL-17C			0.743 (0.628–0.858)	0.742 ± 0.058	0.738 ± 0.044
NTF3, uPA			0.711 (0.595–0.827)	0.712 ± 0.061	0.714 ± 0.014
IL-10, IL-17C			0.715 (0.598–0.833)	0.713 ± 0.067	0.715 ± 0.027
IL-10, uPA			0.715 (0.600–0.831)	0.715 ± 0.069	0.721 ± 0.022
IL-17C, uPA			0.780 (0.669–0.891)	0.778 ± 0.059	0.779 ± 0.035

AUC, area under the curve; CI, confidence interval; CV, cross-validation; FC, fold change; LOOCV, leave-one-out cross-validation; SE, standard error. ^a^Welch’s *t*-test on log2-transformed expression. ^b^Analysis of covariance (ANCOVA) on log2-transformed expression adjusting for age. ^c^Confidence intervals for AUCs estimated by DeLong’s method. ^d^The jackknife standard error estimate. ^e^The standard deviation of cross-validation AUC. ^*^P < 0.05, ^**^P < 0.01.

Based on the age-adjusted ANCOVA results (Supplementary Fig. 2), IL-10 and uPA showed evidence of age-related effects, whereas the other two biomarkers did not exhibit clear age associations. All four proteins remained significantly different between the Parkinson’s disease and HC groups after adjustment for age, with little effect on their overall diagnostic performance. Consistently, ROC analyses adjusted for age yielded equivalent results across all markers (Supplementary Fig. 3). Analyses were not adjusted for sex because of significant differences in sex distribution between the two groups of the new cohort, which may confound the interpretation by attributing group separation largely to sex-related effects.

### Correlation between validated biomarkers and clinical characteristics

**Figure 3:**
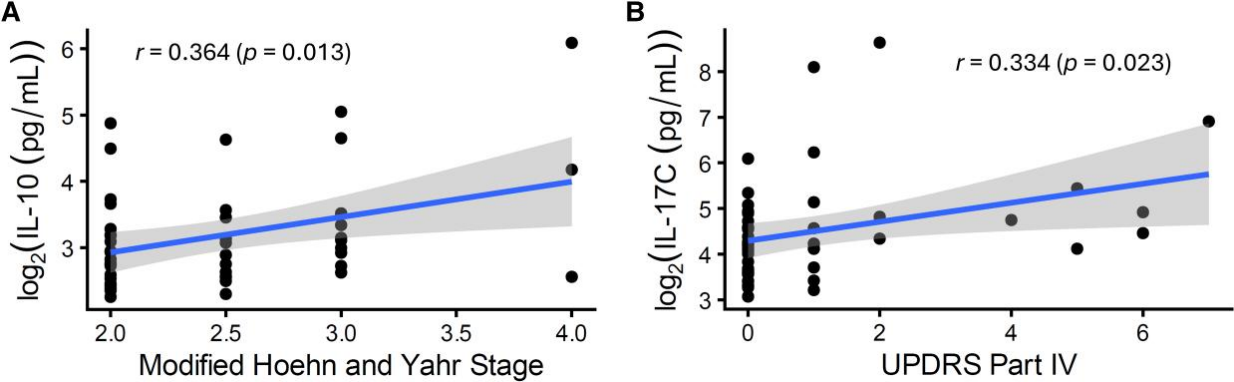
**Associations between plasma protein levels and clinical features of Parkinson’s disease.** (**A**, **B**) Scatter plots showing Pearson correlations between two plasma inflammatory proteins and clinical measures in patients with Parkinson’s disease (n=46). Each data point represents an individual patient, showing the clinical score (on the horizontal axis) and the corresponding log2-transformed plasma protein concentration (on the vertical axis). (**A**) IL-10 levels plotted against the modified Hoehn and Yahr stage, and (**B**) IL-17C levels plotted against UPDRS Part IV scores. Solid lines represent linear regression fits with 95% confidence intervals. Abbreviations: UPDRS part IV, drug complications.

We evaluated whether the absolute concentrations of the four protein biomarkers and their pairwise linear predictors (β-weighted logit values) were associated with motor severity and cognitive function based on clinical measures, including the UPDRS, modified Hoehn and Yahr scale, K-MMSE and CDR (Supplementary Fig. 4). Overall, most biomarkers showed no strong association with the core motor symptomatic index, UPDRS Part III. However, IL-10 and IL-17C exhibited weak but nominal associations with the selected clinical measures, with IL-10 showing an association with the modified Hoehn and Yahr stage and IL-17C with UPDRS Part IV (Fig. 3). Similarly, some multitarget linear predictors incorporating IL-10 and IL-17C showed weak associations with selected clinical measures, including the UPDRS Part IV, modified Hoehn and Yahr stage and SEADL score (Supplementary Fig. 5). Although these associations were not observed uniformly across all clinical indices, they indicate that integrating multiple plasma markers can modestly enhance biomarker performance and capture limited clinical relevance beyond that of single protein analyses.

**Figure 4:**
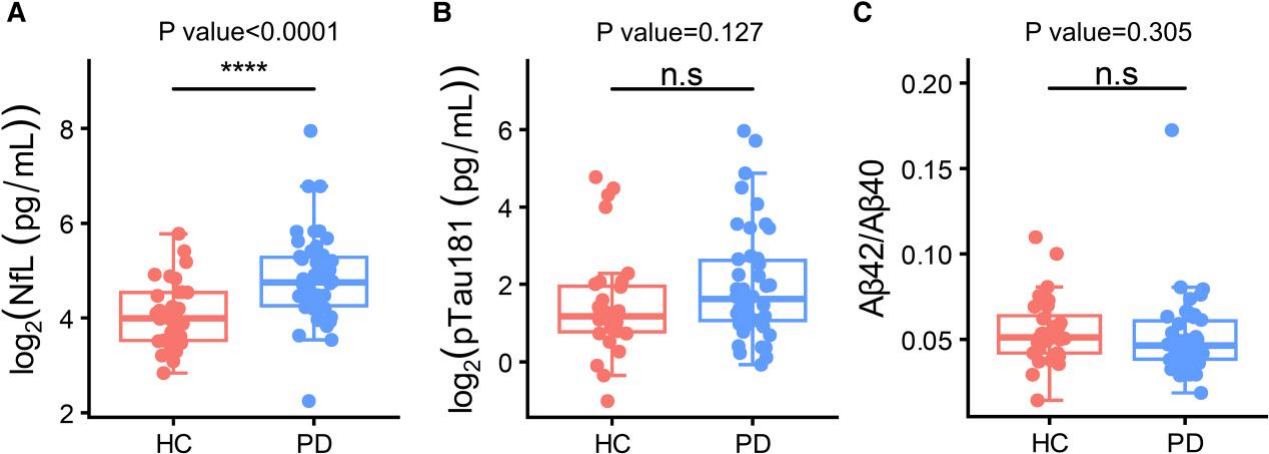
**Plasma levels of NfL, pTau181 and Aβ42/Aβ40 ratio in the Parkinson’s disease and HC groups.** (**A–C**) Box plots showing plasma levels of (left to right) NfL, pTau181 and the Aβ42/Aβ40 ratio measured using the Simoa platform in Parkinson’s disease patients (n=46) and HC (n=33). Each data point represents a sample, showing its log2-transformed absolute plasma concentration. NfL levels were significantly higher in the Parkinson’s disease group (*P* < 0.001), while no significant differences were observed for pTau181 or Aβ42/Aβ40, indicating the absence of Alzheimer’s disease-related pathology in this Parkinson’s disease cohort. *t* values and *P*-values of Welch’s *t*-test: log_2_(NfL): t=−4.211(P<0.001); log_2_(pTau181): t=−1.546(P=0.127); Aβ42/Aβ40: t=1.034(P=0.305). Abbreviations: HC, healthy control; PD, Parkinson’s disease.

Neither the four validated protein biomarkers nor their multitarget linear predictors showed meaningful associations with cognitive measures, including the K-MMSE and CDR (Supplementary Fig. 4). These results indicate that variations in global cognitive performance within this Parkinson’s disease cohort are not captured by the biomarkers examined, suggesting a limited role of these markers in reflecting cognitive impairment. To further investigate whether cognitive changes might instead be attributable to concomitant Alzheimer’s disease pathology, we measured conventional Alzheimer’s disease-related blood biomarkers using the Simoa platform (Fig. 4). While the plasma levels of NfL, a marker of axonal degeneration, were significantly elevated in patients with Parkinson’s disease, no significant differences were observed in plasma pTau181 levels or the Aβ42/Aβ40 ratio. Taken together, these findings suggest that cognitive status in this cohort is unlikely to be driven by Alzheimer’s disease co-pathology.

### Cross-cohort validation of candidate biomarkers using GNPC and UK Biobank data

To evaluate the generalizability of our findings, we performed external validation using two large-scale independent datasets (GNPC and UK Biobank). In the GNPC cohort, the two proteins NTF3 and uPA showed significance, both exhibiting reduced levels in Parkinson’s disease, consistent with the findings in our discovery and validation cohorts (Table 3). While the effect sizes for these markers were relatively modest compared to previously reported Parkinson’s disease-associated biomarkers in the GNPC dataset, the downregulated signal of NTF3 remains noteworthy. This discrepancy in effect size may reflect our more stringent criteria for HC, which excluded individuals with metabolic, inflammatory, psychiatric and neurological comorbidities, in contrast to previous GNPC studies that utilized broader neurological normal controls. This stringent screening represents a deliberate and widely adopted strategy in neurodegenerative biomarker research to maximize disease specificity, as systemic comorbidities can independently alter peripheral protein profiles and introduce false-positive signals unrelated to Parkinson’s disease pathology.^25-27^ Although IL-17C did not show a significant difference in this specific dataset, the overall expression patterns of NTF3 and uPA corroborated our primary findings.

**Table 3:** Differential expression and diagnostic performance of protein biomarkers in the Global Neurodegeneration Proteomics Consortium and the UK Biobank

Global Neurodegeneration Proteomics Consortium (SomaScan®)							
SOMAmer SeqID	Ensembl gene symbol	$n_{HC}$	$n_{PD}$	$n_{Cohort}$	Std. coef.^a^	*P*-value^a^	Up/down^a^
Seq.13723.6	IL-10	2788	277	8	−0.106	0.124	**·**
Seq.2773.50	IL-10	2788	277	8	−0.022	0.750	**·**
Seq.9255.5	IL-17C	2788	277	8	0.013	0.849	**·**
Seq.4145.58	NTF3	2788	277	8	−0.141	0.025	Down
Seq.4158.54	PLAU(uPA)	2788	277	8	−0.184	0.007	Down

UK Biobank (Olink® Explore 3072)							
Protein name	Ensembl gene symbol	$n_{HC}$	$n_{PD}$	$n_{Cohort}$	Std. coef.^b^	*P*-value^b^	Up/down^b^
Interleukin-10	IL-10	16 500	725	1	0.056	0.139	**·**
Interleukin-17C	IL-17C	16 500	725	1	0.082	0.029	Up
Neurotrophin-3	NTF3	16 773	737	1	−0.030	0.432	**·**
Urokinase-type plasminogen activator	PLAU(uPA)	16 817	735	1	−0.098	0.008	Down

Std. coef., standardized coefficient. ^a^ANCOVA on log2-transformed expression adjusting for age, sex and cohort. ^b^ANCOVA on log2-transformed expression adjusting for age and sex.

In a further validation using the UK Biobank, IL-17C and uPA demonstrated nominal significance in the expected direction, whereas NTF3 showed no significant difference between the Parkinson’s disease and HC groups (Table 3). The intrinsic biological heterogeneity and complex comorbidity profiles characteristic of a large-scale population study such as the UK Biobank might have contributed to the attenuated effect sizes. Nevertheless, the consistent significance of IL-17C and uPA across diverse populations underscores their potential as robust indicators of Parkinson’s disease-related pathological alterations.

Despite the modest statistical strength of replication in these external datasets, except for uPA, the consistent directional trends observed for IL-17C, NTF3 and uPA across multiple, heterogeneous populations provide compelling evidence for their biological relevance in Parkinson’s disease.

## Discussion

This study aimed to identify and validate plasma protein biomarkers for Parkinson’s disease using Olink PEA technology. While two previous studies employed Olink PEA for the blood analysis of patients with Parkinson’s disease, both relied solely on relative quantification without absolute quantification-based validation in independent cohorts.^5,11^ Therefore, we analysed plasma from patients with Parkinson’s disease and HC using Olink PEA panels and then validated selected markers using Olink Flex and Luminex platforms for absolute quantification.

Beyond identifying individual protein changes, our analysis of the inter-relationships between the four plasma biomarkers revealed minimal pairwise correlations across the study participants. The only significant correlation was a modest positive association between NTF3 and uPA (Supplementary Fig. 1). Pairwise correlation analyses revealed low inter-marker correlations, which provide statistical support for multivariable modeling by indicating limited multicollinearity, although we acknowledge that assay-related variability may partially contribute to this observation. The cross-platform and cross-cohort replication of consistent directional changes, together with the established roles of these markers in distinct pathophysiological processes—neuroinflammation, neurotrophic signaling and proteostasis—suggests that biological independence is the principal basis for the observed low correlations.

We identified four promising biomarkers: IL-10, IL-17C, NTF3 and uPA. The identification of IL-17C elevation in the plasma of patients with Parkinson’s disease aligns with the role of neuroinflammation in the pathogenesis of Parkinson’s disease. Although direct evidence linking IL-17C to neurodegeneration or neuroinflammation remains limited, emerging studies reveal key mechanistic connections. IL-17C strongly affects macrophage polarization, which may influence microglial function in the central nervous system.^28^ Furthermore, excessive IL-17C levels can lead to tissue damage and the secretion of proinflammatory cytokines.^29^ The observed downregulation of NTF3 in Parkinson’s disease is also noteworthy. NTF3 is a neurotrophic factor essential for the survival and function of dopaminergic neurons. Previous studies exploring the neuroprotective effects of NTF3 in an MPTP-based animal model of Parkinson’s disease have shown that this protein enhances the survival of dopaminergic neurons and improves motor function.^30^ In addition, transcriptomic analysis of an MPTP/probenecid-based mouse model of slowly progressing Parkinson’s disease identified early downregulation of *Ntf3* in the substantia nigra during the pre-neurodegenerative phase.^31^ However, direct clinical evidence demonstrating reduced plasma NTF3 levels in patients with Parkinson’s disease remains limited.

The uPA system is crucial in CNS neuroinflammation, primarily because of its interaction with microglial cells. uPA receptors in resident microglial cells are significantly upregulated during neuroinflammatory conditions.^32^ Additionally, recent proteomics studies show reduced plasma uPA levels in patients with Parkinson’s disease.^11^

The biological mechanisms underlying the observed plasma alterations in IL-17C, NTF3 and uPA concentrations are consistent with the known pathophysiology of Parkinson’s disease. Elevated IL-17C levels are concordant with mounting evidence of Th17-mediated immune dysregulation in Parkinson’s disease. Sustained IL-17 signaling promotes microglial and astrocytic activation, blood–brain barrier disruption and the release of neurotoxic mediators, all of which contribute to the loss of dopaminergic neurons.^33-35^ Downregulation of NTF3 levels has been reported in Parkinson’s disease-relevant brain areas, and experimental evidence suggests that the administration of exogenous NTF3 can rescue dopaminergic neurons in both animal *in vivo* and *in vitro* models.^30,36^ Lower plasma uPA concentrations are interpretable within the context of impaired extracellular proteostasis. The uPA–plasmin system is involved in the degradation of extracellular protein aggregates, and dysregulation of plasminogen activators and their inhibitors has been linked to α-synuclein pathology and neuroinflammation in models of Parkinson’s disease.^37,38^ Taken together, these findings suggest that our biomarker panel captures complementary aspects of the pathophysiology of Parkinson’s disease, including neuroinflammation via IL-17 family signaling, withdrawal of dopaminergic trophic support via NTF3, and weakened proteolytic aggregate clearance via uPA.

An important methodological consideration in this study is the limitation of Olink NPX values, which represent relative, protein-specific normalized measurements. While this approach is well-suited for high-throughput discovery, it constrains direct quantitative integration across multiple proteins and limits robust multitarget modeling. To address this, we validated candidate biomarkers using absolute protein concentrations measured by Olink Flex and Luminex platforms. This strategy enabled the application of multivariable logistic regression and provided a more clinically interpretable framework for assessing combined biomarker performance.

Using absolute quantification, the levels of all four proteins remained significantly different between the Parkinson’s disease and control groups, confirming the robustness of the discovery-stage findings. However, the diagnostic performance of individual biomarkers was modest. Although multitarget models incorporating IL-17C with either NTF3 or uPA showed significant improvements over single-protein models, their overall performance remained moderate. These findings suggest that multitarget inflammatory models may offer the potential for more comprehensive diagnostic approaches for Parkinson’s disease, rather than serving as definitive standalone diagnostic tools.

Age-adjusted analyses demonstrated that although IL-10 and uPA exhibited age-related trends, all four biomarkers remained significantly different between Parkinson’s disease and HC groups after adjustment, with minimal effect on diagnostic performance. Adjustment for sex was not performed in the validation cohort due to substantial sex imbalance; however, the analyses in the sex-matched discovery cohort suggest that sex-related effects are unlikely to substantially alter the observed biomarker differences.

Limited associations between inflammatory biomarkers and clinical measures were observed. Neither individual proteins nor multitarget models showed meaningful correlations with core motor severity as assessed by UPDRS Part III. Weak associations were observed between IL-10 and disease stage, as well as between IL-17C and drug complications assessed by UPDRS Part IV. Some multitarget combinations incorporating IL-10 and IL-17C also showed nominal associations with functional measures, such as the SEADL score. These findings suggest that inflammatory biomarkers may reflect specific aspects of disease burden or treatment-related complications rather than overall motor severity. However, the discriminative capacity of the combination IL-17C with uPA remains within the range reported for current blood-based proteomic panels in Parkinson’s disease and may serve as a practical screening adjunct to confirmatory neuroimaging, particularly in primary and secondary care settings where cost-effective triage tools are needed.

Importantly, neither individual biomarkers nor multitarget combinations were associated with global cognitive measures, including K-MMSE and CDR. To assess whether cognitive differences shown in Table 1 might reflect concomitant Alzheimer’s disease pathology, we measured established Alzheimer’s disease-related plasma biomarkers. We analysed the plasma levels of pTau181, Aβ42, Aβ40 and NfL. Given the established sensitivity of NfL as a marker of neurodegeneration, we further explored whether combining NfL with the inflammatory candidates would enhance diagnostic discrimination. Among the NfL-inclusive models, NfL paired with uPA achieved the highest performance (unadjusted AUC: 0.853, age-adjusted AUC: 0.868), surpassing the best inflammatory combination of IL-17C and uPA (AUC: 0.780). Age adjustment uniformly improved NfL-based model performance, likely reflecting the known age-dependent increase in the serum concentration of this marker. However, because NfL levels are broadly elevated across neurodegenerative conditions, such as atypical parkinsonism, Alzheimer’s disease and amyotrophic lateral sclerosis,^39,40^ their contribution to differential diagnosis beyond a comparison of Parkinson’s disease versus control remains uncertain. Therefore, we report these NfL-inclusive analyses as supplementary findings (Supplementary Fig. 6) while retaining the inflammatory marker panel as our primary diagnostic model, given its potential to capture PD-relevant pathophysiological signals with greater disease specificity. Despite some studies suggesting a connection between pTau181 and cognitive decline in Parkinson’s disease, as well as its increased presence in the plasma of patients with Parkinson’s disease, our analysis did not show any significant differences in plasma pTau181 levels or the Aβ42/Aβ40 ratio between the groups.^41-43^

This study has several strengths, notably using the sensitive Olink PEA technology for multiplex protein analysis with rigorous statistical methods. The use of [^18^F]-FP-CIT PET imaging to confirm the diagnosis of Parkinson’s disease within the patient cohort further bolsters the reliability of the results. [^18^F]-FP-CIT PET is known for its exceptional sensitivity and specificity in diagnosing Parkinson’s disease and distinguishing it from other parkinsonian syndromes.^44,45^ Our application of multivariable logistic regression using absolute protein concentrations, following biomarker discovery based on relative Olink NPX measurements, represents a systematic approach for multitarget biomarker validation. By confirming Olink-identified plasma biomarkers using absolute quantification platforms, such as Luminex and Olink Flex, this strategy strengthens analytical robustness and supports their relevance for clinical biomarker development.

External validation using GNPC and UK Biobank proteomic datasets provided modest and directionally consistent support for selected biomarkers. In the GNPC dataset, NTF3 and uPA showed nominal significance, whereas in the UK Biobank dataset, IL-17C and uPA demonstrated modest associations in the expected direction. The attenuated effect sizes observed in these cohorts likely reflect differences in cohort composition, disease definition, comorbidity burden and analytical platforms. Nevertheless, the recurrence of directionally consistent signals across independent populations supports the biological relevance of these inflammatory markers in Parkinson’s disease.

Several limitations should be considered when interpreting the current results. The relatively small sample size may limit the generalizability of our results and the statistical power to detect subtle biomarker variations among Parkinson’s disease subtypes. Future investigations should include larger participant groups to strengthen the findings and provide a more detailed understanding of the roles of biomarkers across various Parkinson’s disease subtypes. Furthermore, the cross-sectional nature of the study design prevented the establishment of causal links between inflammatory markers and other clinical variables. Longitudinal studies with larger sample sizes are necessary to confirm the identified biomarkers and investigate their potential roles in the progression and management of Parkinson’s disease. Additionally, the study faced a limitation due to the significant sex ratio imbalance among participants, which restricted comprehensive covariate adjustment and may have affected the outcomes of ROC analyses.

In conclusion, our study has identified promising protein biomarkers, particularly IL-17C, NTF3 and uPA, in the plasma of patients with Parkinson’s disease. The multitarget combination of IL-17C and uPA showed diagnostic potential for distinguishing patients with Parkinson’s disease from HC (sensitivity = 0.783, specificity = 0.788, AUC = 0.780). These findings highlight the role of neuroinflammation in Parkinson’s disease and underscore the potential of plasma-based protein biomarkers for improving the diagnosis of Parkinson’s disease. Future research should validatethese findings in larger cohorts, investigate biological mechanisms and explore these biomarkers for therapeutic development.

## Supplementary Material

fcag282_Supplementary_Data

## Data Availability

The data that support the findings of this study are available from the corresponding author on reasonable request. All statistical analysis code used in this study is available at https://github.com/KBRI-Neuroinformatics/PD-multi-target-panel.
