# Supplementary material for: Identification of a robust multitarget protein panel for Parkinson’s disease via absolute quantification and large-scale external replication
Source: Brain Commun. 2026 Jul 16;8(4):fcag282. doi: 10.1093/braincomms/fcag282 (PMC13403565; doi:10.1093/braincomms/fcag282)
Supplement: fcag282_Supplementary_Data [file fcag282_supplementary_data.docx]

**Supplementary Materials**

**
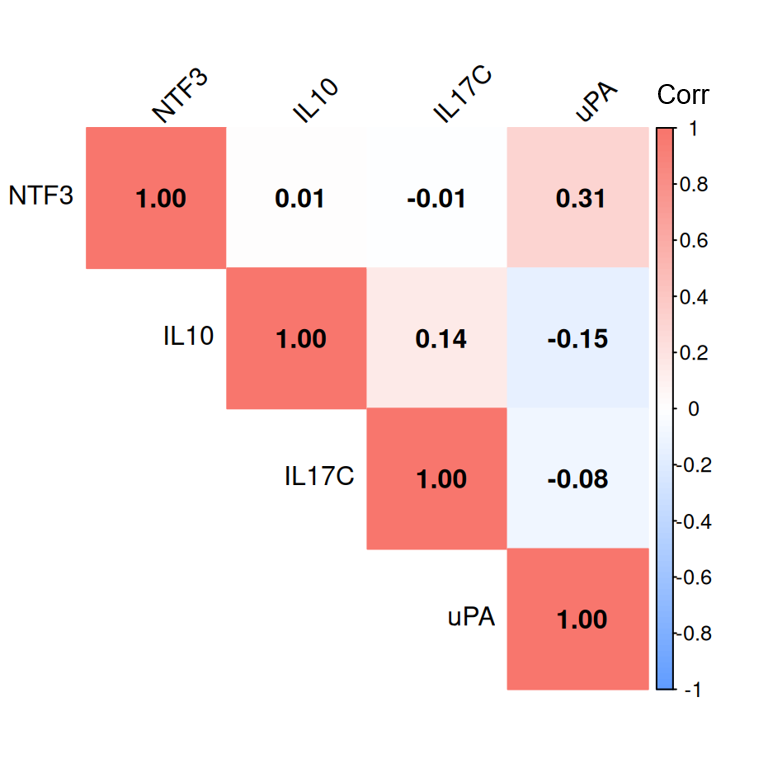
**

**Supplementary Figure 1. Pairwise correlations among plasma biomarkers across all participants.** Heatmap showing Pearson correlation coefficients between absolute plasma concentrations of IL-10, IL-17C, NTF3, and uPA calculated across all study participants, including both patients with Parkinson’s disease ($n=46$) and HC ($n=33$). With the exception of a modest correlation between NTF3 and uPA, minimal pairwise correlations were observed, indicating limited multicollinearity and supporting the use of multivariable logistic regression for combined biomarker modeling. Pearson correlation coefficients (r) and p-values: NTF3–IL10: $r=0.013 (p=0.909)$; NTF3–IL17C: $r=-0.008 (p=0.942)$; NTF3–uPA: $r=0.311 (p=0.005)$; IL10–IL17C: $r=0.140 (p=0.218)$; IL10–uPA: $r=-0.152 (p=0.180)$; IL17C–uPA: $r=-0.084 (p=0.463)$.

**
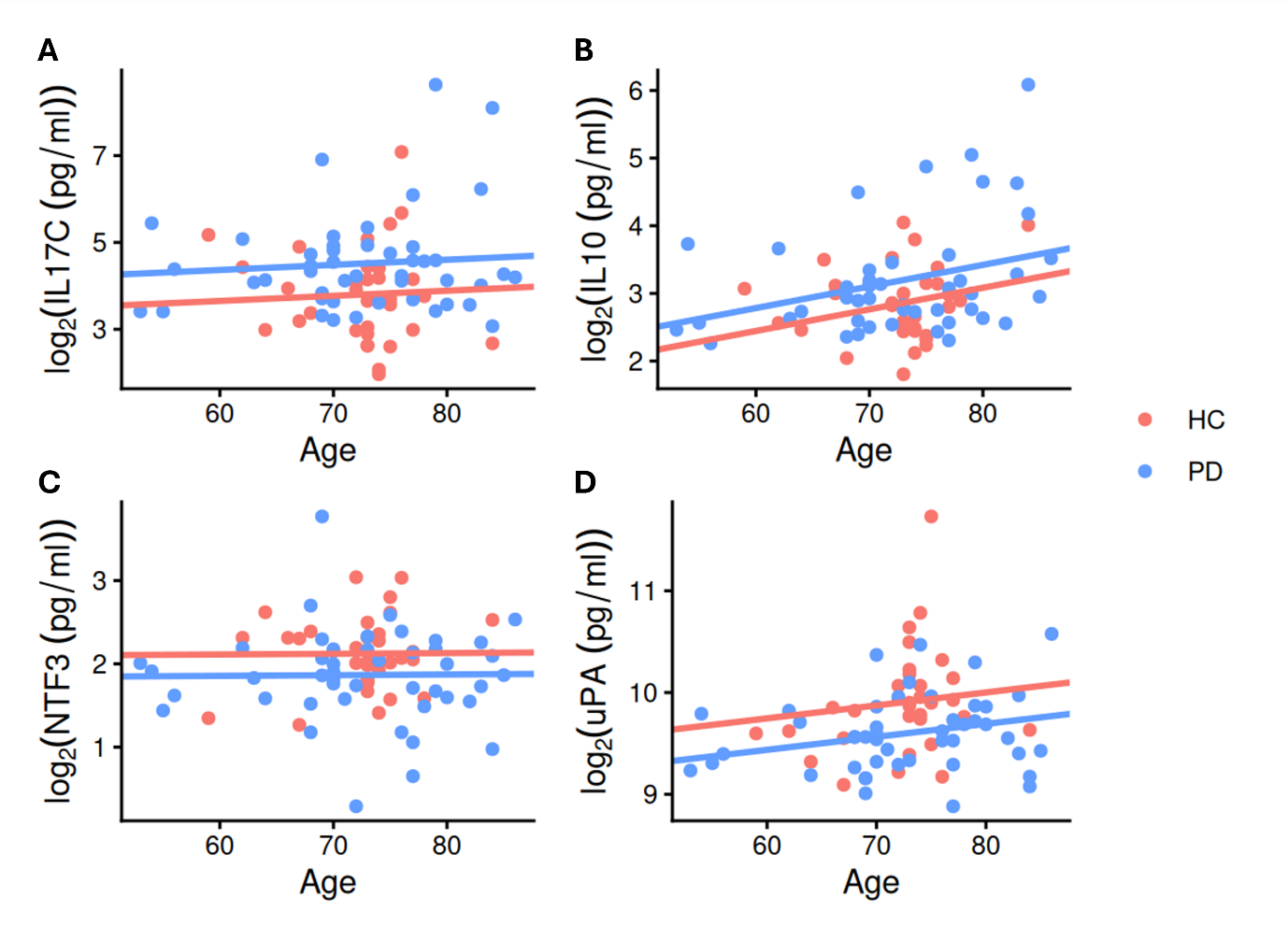
**

**Supplementary Figure 2. Age-adjusted distributions of inflammatory biomarkers in Parkinson’s disease and HC.** Scatter plots showing plasma levels of IL-17C, IL-10, NTF3, and uPA as a function of age in patients with Parkinson’s disease ($n=46$) and HC ($n=33$). Each data point represents an individual patient, showing the age (on the horizontal axis) and the corresponding log2-transformed plasma protein concentration (on the vertical axis). Solid lines indicate age-adjusted trends for each clinical group estimated by ANCOVA. Despite evidence of age-related effects for selected proteins, all four biomarkers remained differentially expressed between Parkinson’s disease and HC after adjustment for age. *F* values and p-values of ANCOVA: IL17C ($F=7.461, p=0.008$), IL10 ($F=4.597, p=0.035$), NTF3 ($F=4.683, p=0.034$), and uPA ($F=9.778, p=0.003$).

Abbreviations: HC, healthy control; PD, Parkinson’s disease.

**
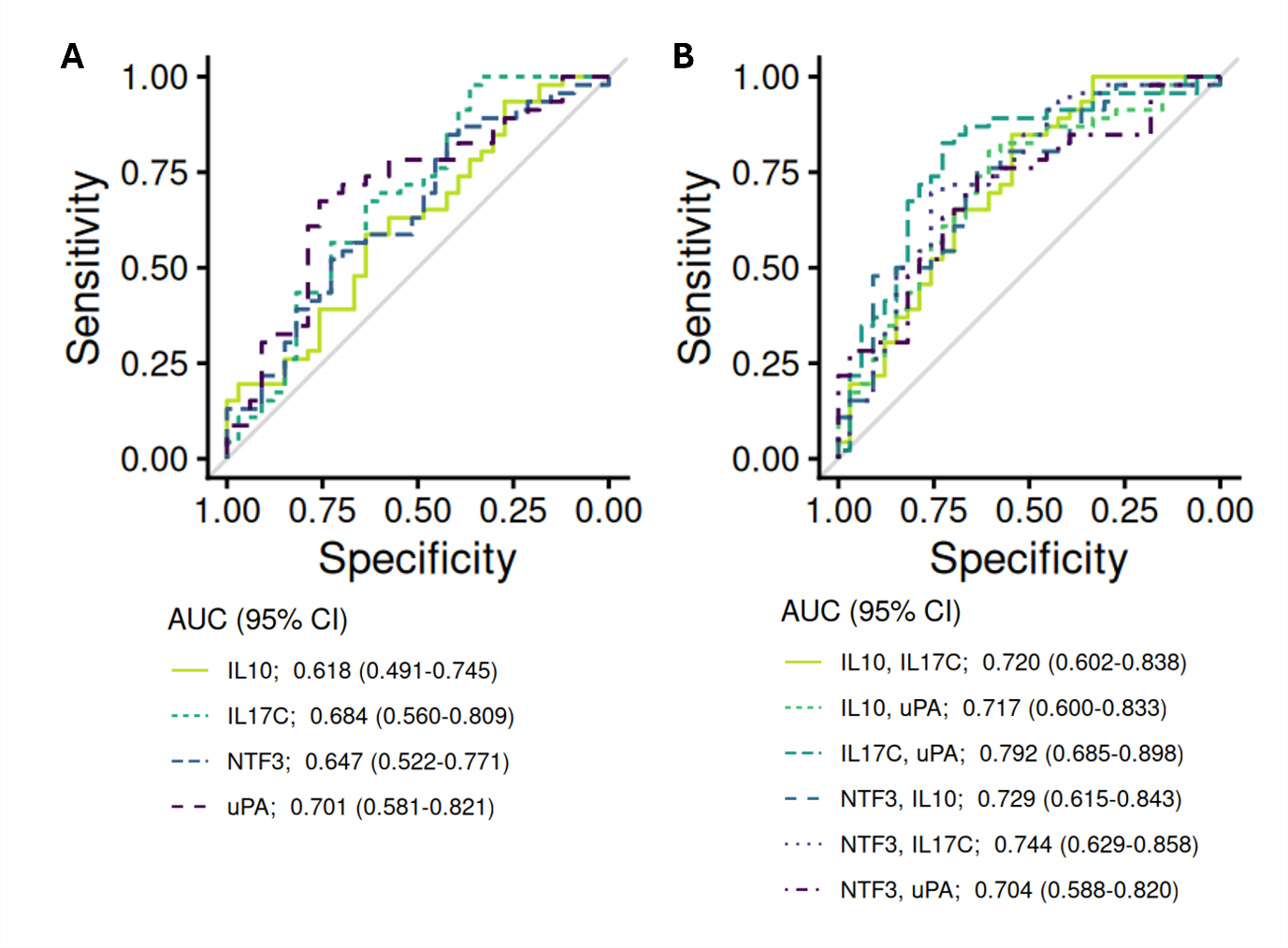
**

**Supplementary Figure 3. Age-adjusted diagnostic performance of inflammatory biomarkers.** ROC curves showing the diagnostic performance of four individual biomarkers and their pairwise multivariable combinations based on age-adjusted logistic regression models in patients with Parkinson’s disease ($n=46$) and HC ($n=33$). ROC analyses adjusted for age yielded performance comparable to unadjusted models (Figure 2C), indicating that age had minimal impact on the discriminative ability of these biomarkers.

Abbreviations: AUC, area under the curve; CI, confidence interval; ROC, Receiver operating characteristic.

**
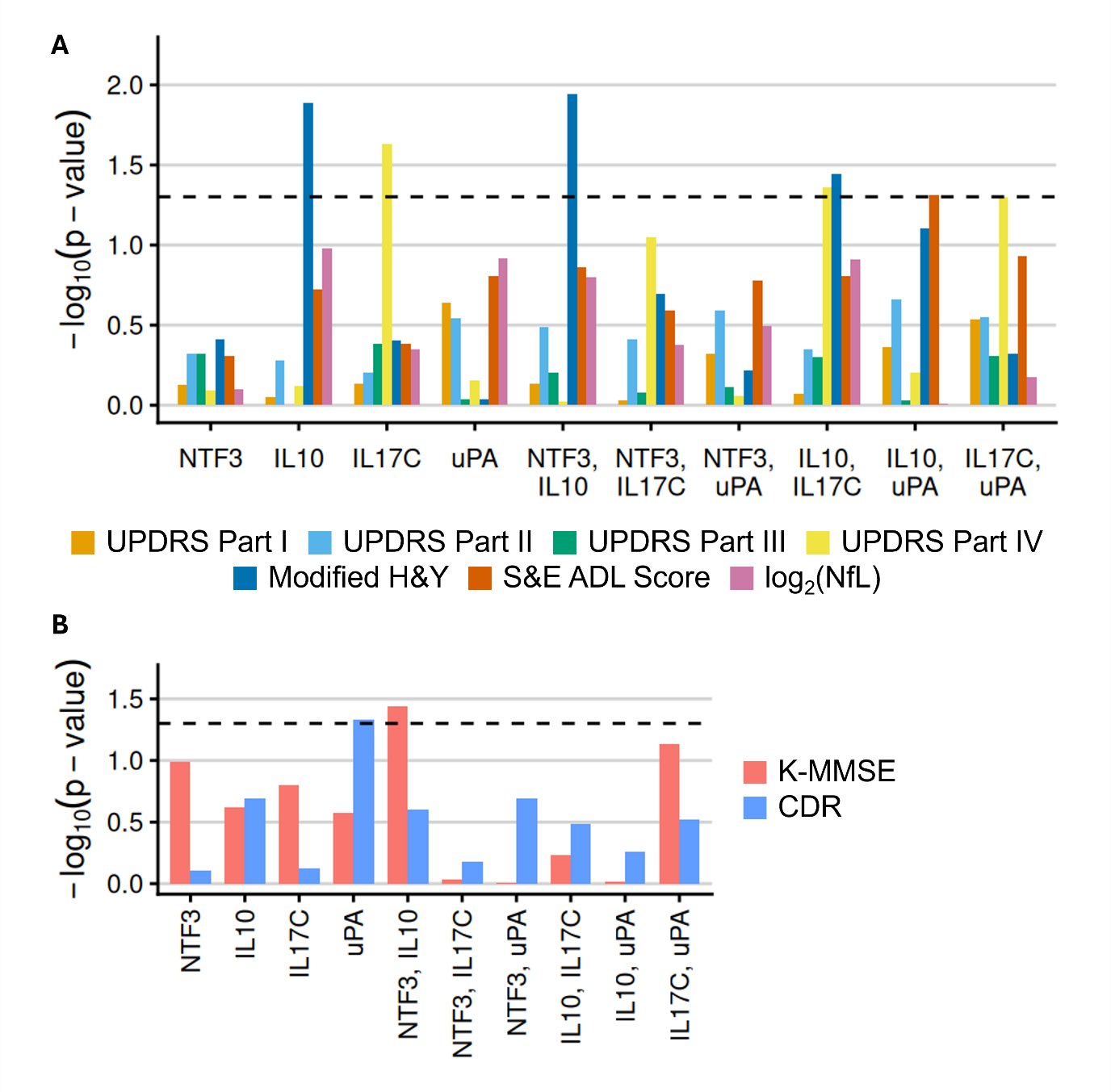
**

**Supplementary Figure 4. Associations between inflammatory biomarkers, neurodegeneration markers, and clinical measures in Parkinson’s disease (**$\boldsymbol{n=46}$**). (A)** Bar plots showing −log_10_(p-values) for Pearson correlations between absolute plasma levels of four inflammatory biomarkers (IL-10, IL-17C, NTF3, uPA), and their multivariable combinations with clinical measures including UPDRS subscales, the modified Hoehn & Yahr stage, the SEADL score, and plasma NfL levels. **(B)** Bar plots showing −log_10_(p-values) for Pearson correlations between the same biomarkers and biomarker combinations with cognitive measures, including K-MMSE and CDR. Dashed lines indicate nominal significance thresholds.

Abbreviations: CDR, clinical dementia rating; K-MMSE, Korean mini-mental state examination; modified H&Y, modified Hoehn and Yahr stage; S&E ADL, Schwab and England activities of daily living; UPDRS, unified Parkinson's disease rating scale; UPDRS part I, behavior and emotion; UPDRS part II, activities of daily living; UPDRS part III, motor scale; UPDRS part IV, drug complications.

**
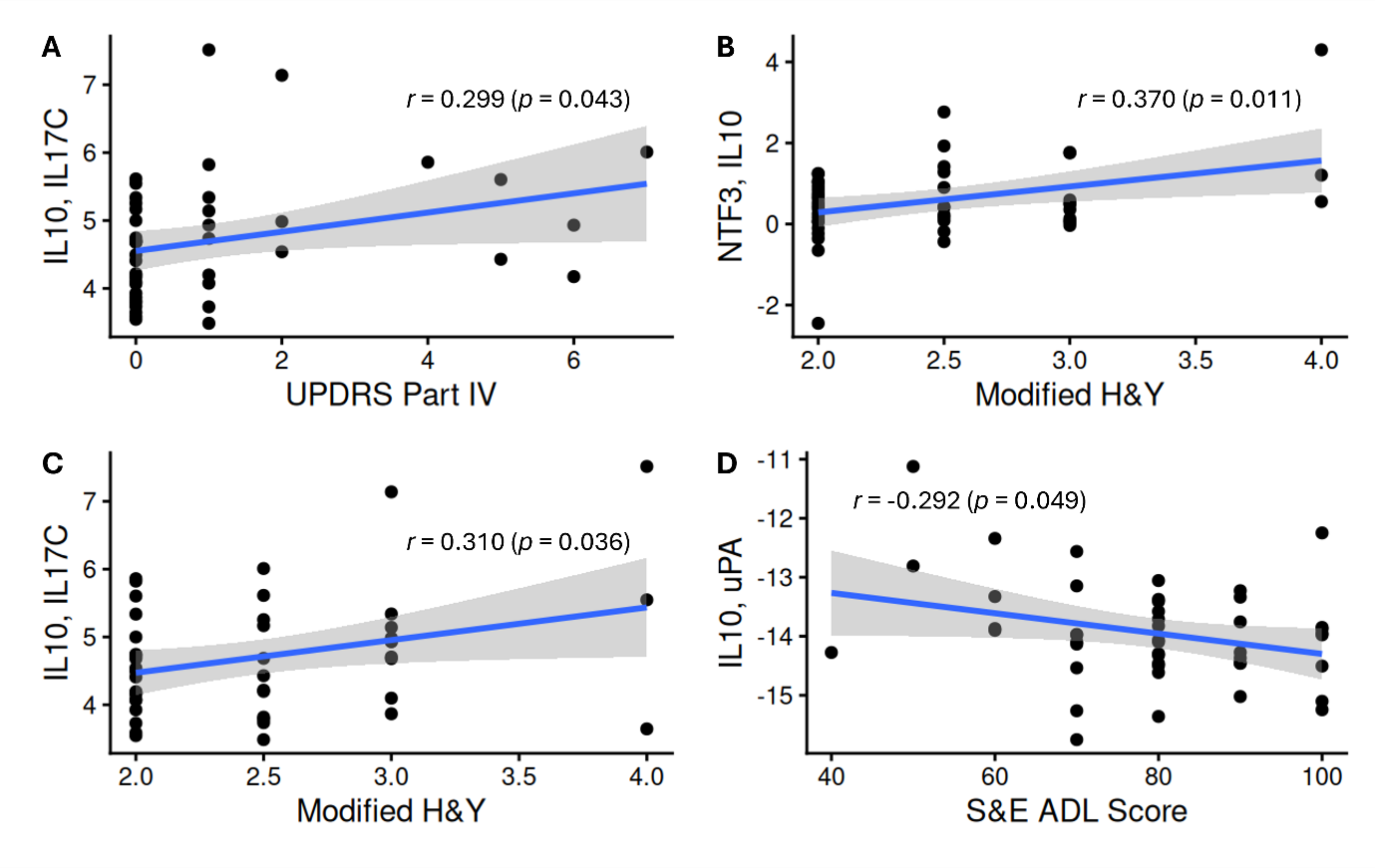
**

**Supplementary Figure 5. Associations between multi-target linear predictors and clinical measures in Parkinson’s disease (**$\boldsymbol{n=46}$**).** Scatter plots showing Pearson correlations between selected pairwise linear predictors derived from absolute protein concentrations and clinical measures, including UPDRS Part IV, the modified Hoehn & Yahr stage, and the SEADL score. Each data point represents an individual patient, showing the clinical measures (on the horizontal axis) and the corresponding linear predictors (on the vertical axis). Solid lines represent linear regression fits with 95% confidence intervals. Linear predictors (β-weighted logit values): NTF3 & IL10 = −1.222·log₂(NTF3) + 0.902·log₂(IL10); NTF3 & IL17C = −1.051·log₂(NTF3) + 0.648·log₂(IL17C); NTF3 & uPA = −0.690·log₂(NTF3) − 1.476·log₂(uPA); IL10 & IL17C = 0.599·log₂(IL10) + 0.619·log₂(IL17C); IL10 & uPA = 0.725·log₂(IL10) − 1.693·log₂(uPA); IL17C & uPA = 0.695·log₂(IL17C) − 1.853·log₂(uPA).

Abbreviations: modified H&Y, modified Hoehn and Yahr stage; S&E ADL, Schwab and England activities of daily living; UPDRS, unified Parkinson's disease rating scale; UPDRS part IV, drug complications.

**
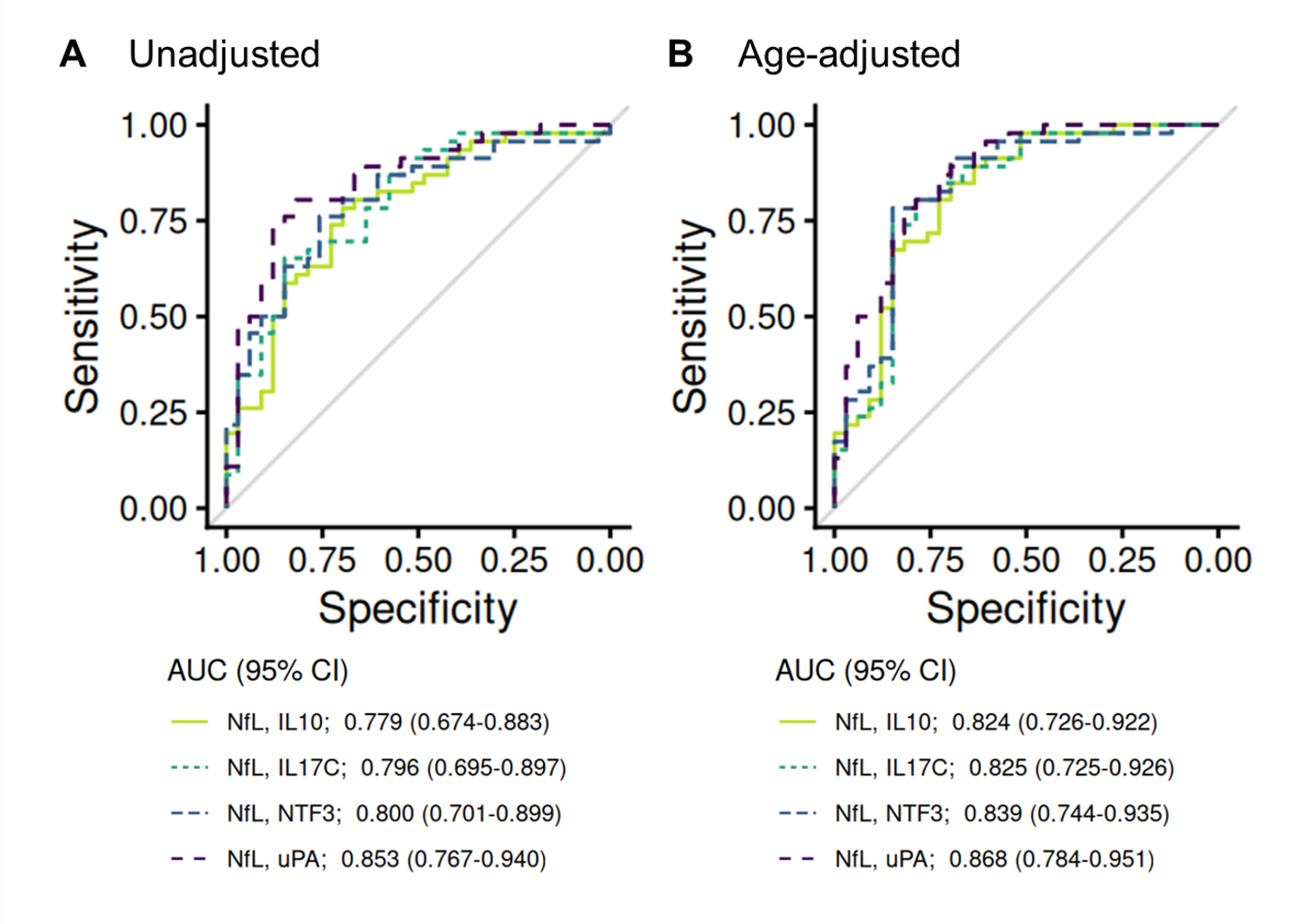
**

**Supplementary Figure 6. Diagnostic performance of multi-target models incorporating NfL.** ROC curves showing the diagnostic performance of logistic regression models combining NfL with individual inflammatory biomarkers (IL-10, IL-17C, NTF3, and uPA) to distinguish patients with Parkinson’s disease (n=46) from HC (n=33), (A) without covariate adjustment and (B) with age adjustment. The NfL + uPA combination yielded the highest AUC in both the unadjusted (0.853; 95% CI: 0.767–0.940) and age-adjusted (0.868; 95% CI: 0.784–0.951) models.

Abbreviations: AUC, area under the curve; CI, confidence interval; ROC, Receiver operating characteristic.

**Supplementary Table 1. Summary of statistical assumption checks and statistical tests.**

| **Discovery Cohort** | **Parkinson’s**  **Disease**  **(**$\boldsymbol{n}\boldsymbol{=12}$**)** | **HC**  **(**$\boldsymbol{n}\boldsymbol{=15}$**)** | **Test for** | **Statistical Test** |
| --- | --- | --- | --- | --- |
| Age (year) | p=0.034^a^ * | p=0.776^a^ | Difference in means | Mann-Whitney U test |
| Gender (F:M) | 5:7  (5.78:6.22) | 8:7  (7.22:7.78) | Independence between two categorical variables | Chi-square test  (every expected frequency > 5) |
| K-MMSE  total score | p=0.006^a^ ** | p=0.160^a^ | Difference in means | Mann-Whitney U test |
| **Validation Cohort** | **Parkinson’s**  **Disease**  **(**$\boldsymbol{n}\boldsymbol{=46}$**)** | **HC**  **(**$\boldsymbol{n}\boldsymbol{=33}$**)** | **Test for** | **Statistical Test** |
| Gender (F:M) | 18:28  (26.2:19.8) | 27:6  (18.8:14.2) | Independence between two categorical variables | Chi-square test  (every expected frequency > 5) |
| Any Variable | $n>30$ | $n>30$ | Difference in means | Welch’s t-test |
| Any Variable | $n>30$ | $n>30$ | Difference in adjusted means | ANCOVA |
| Any Variable | $n>30$ | $n>30$ | linear association between two continuous variables | Pearson’s Correlation |
| **GNPC** | **Parkinson’s**  **Disease**  **(**$\boldsymbol{n=277}$**)** | **HC**  **(**$\boldsymbol{n=2,788}$**)** | **Test for** | **Statistical Test** |
| Any Variable | $n>30$ | $n>30$ | Difference in adjusted means | ANCOVA |
| **UK Biobank** | **Parkinson’s**  **Disease**  **(**$\boldsymbol{n=737}$**)** | **HC**  **(**$\boldsymbol{n=16,817}$**)** | **Test for** | **Statistical Test** |
| Any Variable | $n>30$ | $n>30$ | Difference in adjusted means | ANCOVA |

Note: Data are presented as p-value, frequency (expected) or number of samples (*n*).

Abbreviations: HC, healthy control; K-MMSE, Korean mini-mental state examination.

^a^Shapiro-Wilk test.
